# Cardiovascular imaging in cardio-oncology

**DOI:** 10.1007/s11604-024-01636-x

**Published:** 2024-08-29

**Authors:** Nagara Tamaki, Osamu Manabe, Kenji Hirata

**Affiliations:** 1 Kyoto College of Medical Science, Sonobe, Kyoto Japan; 2 Department of Radiology, Kyoto Prefectural University of Medicine, Kyoto, Japan; 3 Department of Radiology, Jichi Medical University Saitama Medical Center, Saitama, Japan; 4 Department of Diagnostic Imaging, Faculty of Medicine, Hokkaido University, Sapporo, Japan

**Keywords:** Cardiac imaging, Cancer, Cardio-oncology, PET, Molecular imaging

## Abstract

Advances in cancer treatment have improved in patient survival rate. On the other hand, management of cardiovascular complications has been increasingly required in cancer patients. Thus, cardio-oncology has attracted the attention by both oncologists and cardiologists. Cardiovascular imaging has played a key role for non-invasive assessment of cardiovascular alterations complimentary to biomarkers and clinical assessment. Suitable imaging selection and interpretation may allow early diagnosis of cardiovascular injury with potential implications for therapeutic management and improved outcomes after cancer therapy. Echocardiography has been commonly used to evaluate cardiac dysfunction in cardio-oncology area. Cardiac CT is valuable for assessing structural abnormalities of the myocardium, coronary arteries, and aorta. Molecular imaging has an important role in the assessment of the pathophysiology and future treatment strategy of cardiovascular dysfunction. Cardiac MRI is valuable for characterization of myocardial tissue. PET and SPECT molecular imaging has potential roles for quantitative assessment of cardiovascular disorders. Particularly, FDG-PET is considered as an elegant approach for simultaneous assessment of tumor response to cancer therapy and early detection of possible cardiovascular involvement as well. This review describes the promising potential of these non-invasive cardiovascular imaging modalities in cardio-oncology.

## Introduction

Cancer and cardiovascular disease are the leading causes of death in most developed countries. Cancer and cardiovascular disease are closely related from both scientific and clinical perspectives. The likelihood of developing most types of cancer and cardiovascular disease increases with age, and thus, older individuals with cancer are more likely to have high risk of cardiovascular disease. Recent dramatic advances in cancer treatment, including targeted molecular therapy, immunotherapy, and radiotherapy, have dramatically increased the number of cancer survivors. Cancer treatments may often have its characteristic side effects, particularly in cardiovascular system. Thus, management of cardiovascular complications in cancer survivors is increasingly focused [1]. This oncology–cardiology combined area is called either onco-cardiology [2, 3] or cardio-oncology [4]. We used cardio-oncology in this review since the latter term seems to be more internationally applied recently. Cardio-oncology has gained attention by both cardiologists and oncologists. More recently, radiologists have played important roles for suitable image selection and interpretations for assessing cardiovascular complications. In addition, radiology specialists may provide appropriate radiotherapy planning with reduced radiation dose to cardiovascular system. Mutual work among these specialists on pathophysiology analysis and treatment strategy is valuable [2–5]. These specialists should collaborate in the diagnosis and treatment of cancer patients with cardiovascular disease in various clinical settings.

This review summarizes the definitions of the most common cardiovascular complications and the latest findings of cardiotoxicity for cancer therapy, including targeted chemotherapy and radiotherapy. In addition, appropriate non-invasive image analysis for assessing important insights in the early detection and monitoring cardiotoxicity is described.

## Definitions of cardiac dysfunction

The most common cardiotoxicity after cancer therapy is cardiac dysfunction resulting in heart failure. It is defined by the position papers using decrease in left ventricular ejection fraction (LVEF) and or global longitudinal strain following cancer therapy from European Society of Cardiology (ESC) [6], American Society of Echocardiography (ASE) [7], American Society of Clinical Oncology (ASCO) [8], and European Society of Medical Oncology (ESMO) [9].

Recent guidelines released from ESC in cardio-oncology have provided all the healthcare professionals the instruments to take care of oncologic patients before, during and after cancer therapy as far as the cardiovascular system is concerned [4]. These include clear indications regarding how to select and appropriate use of different imaging modalities in various clinical circumstances [4].

Before initiating cancer treatment, baseline imaging is crucial for several reasons. Establishing a baseline helps in identifying any pre-existing cardiovascular conditions that might influence treatment decisions. Baseline imaging can also help stratify patients according to their risk for developing treatment-related cardiotoxicity. It provides essential information for planning cancer treatment, ensuring that any necessary modifications can be made to minimize cardiac risks. Echocardiography is widely used for baseline assessment of cardiac function and structure. Cardiac MRI provides detailed information on cardiac anatomy and function. These modalities are useful for comprehensive baseline assessment [4, 6–9].

## Cardiotoxicity of specific chemotherapies

Anthracyclines (e.g., doxorubicin, daunorubicin) are widely used as the prototype of cardiotoxic cancer therapy of leukemia and various solid tumors [10]. It is well known that anthracyclines are associated with a significant risk of cardiotoxic side effects. Left ventricular dysfunction and consecutive heart failure are as the most important form considering their profound impact on morbidity and mortality after cancer therapy [11].

Antimetabolites are considered to disrupt the formation of DNA and RNA. Notable antimetabolites include 5-fluorouricil (5-FU), capecitabine, cytarabine, gemcitabine, methotrexate, and hydroxyurea. These drugs are frequently used in the treatment of leukemia and cancers of the ovary, breast, gastrointestinal tract, and other solid tumors. The most common issues reported in cardiac field are myocardial ischemia, angina, chest discomfort, and changes in the electrocardiogram (ECG), such as ST-segment and T-wave alterations [12]. The harmful cardiac effects of 5-FU and capecitabine are believed to be caused by several factors, including damage to the endothelial cells leading to thrombosis, increased metabolic activity resulting in energy shortage and ischemia. Oxidative stress leading to cell damage, coronary artery spasm, and reduced oxygen delivery by red blood cells also leads to myocardial ischemia [13].

During the past 2 decades, targeted therapeutics have been introduced and increasingly applied in cancer therapy. The first evidence of significant cardiotoxicity was found for trastuzumab, an inhibitor of human epidermal growth factor receptor 2 (HER2) that is commonly used in the treatment of HER2-positive breast cancer [14]. Significant progress in the development of new drugs has led to the increasing use of targeted therapeutics for cancer therapy with great improvements in morbidity and mortality. Such increased applications of targeted therapeutics have unmasked various forms of side effects, including severe cardiovascular toxicities [15].

Immune checkpoint inhibitor therapy induces an anti-tumor immune reaction by blocking immune-inhibitory signaling via the programmed death 1 (PD1) pathways [16]. The survival rate of cancer patients after this therapy has been greatly improved, particularly for melanoma and non-small cell lung cancer. On the other hand, this therapy is associated with the risk of autoimmune-triggered immune-related adverse events (irAEs), including a significant risk of cardiotoxicity. The most recognized form is myocarditis which may cause cardiogenic shock and severe arrhythmia [16, 17]. Cardiac irAEs are often treated with immunosuppressive therapy. The second most common cardiovascular complication of immune checkpoint inhibitor therapy is pericardial disease, such as pericardial effusion and pericarditis.

There are number of other targeted therapeutics which have been applied for specific cancers. Such new therapy has indicated great improvements in prognosis in cancer, but also suggested increased risk of severe cardiovascular toxicities [18–23]. Wide applications are seen with new progress in the field of targeted therapeutics together with the growing number of long-term survivors after oncology therapy. On the other hand, cardiotoxic side effects have become essential for the best and suitable treatment strategy in cardio-oncology.

## Cardiovascular complications after radiotherapy

Radiotherapy is an increasingly applied cancer-therapeutic method. When applying radiotherapy to malignant tumors in left breast cancer and esophageal cancer, cardiotoxicity can be caused by high dose of radiation delivered to the myocardium. The likelihood and intensity of cardiovascular complications rise with an increase in the radiation dose, the size of the area exposed, and radiation exposure at a younger age [24]. The risk also grows the longer the time of radiotherapy, with use of additional chemotherapy, and when other metabolic risk factors like hypertension, smoking, obesity, and diabetes are present [24]. Careful follow-up care of cancer patients is particularly important after radiotherapy combined with chemotherapy.

Radiotherapy is well known to have significant cardiovascular complications, such as pericarditis and long-term complications, such as restrictive or constrictive pericarditis. Approximately 35% of cancer patients undergo radiotherapy within 1 year after diagnosis [25]. Radiation therapy to treat tumors near the heart increases the risk of developing radiation-induced valvular heart disease. Moreover, patients who have previously undergone mediastinal radiation therapy may face a higher risk of complications and death following valve surgery [24]. Radiotherapy-related myocarditis and vasculitis are known as acute complication, but its incidence has decreased due to dose fractioning [26]. Suitable diagnosis is considered valuable for identifying and monitoring such early complications, using blood tests and molecular imaging if it is required.

Long-term complications of radiotherapy involving the heart are cardiac fibrosis and coronary atherosclerosis. Such complications may arise with a latency of several decades after exposure to radiation [27]. These long-term complications include coronary artery disease, valvular disease, and diastolic dysfunction. Radiation during childhood and concomitant exposure to anthracyclines is associated with a significant increased risk for cardiac complications [28]. Therefore, careful follow-up of young cancer patients is particularly important after radiotherapy.

## Image assessment of cardiovascular toxicities after cancer therapy

Appropriate diagnosis and assessment of cardiovascular toxicities plays an important role for cardio-oncology. There are number of methods commonly used for detecting cardiovascular disease and assessing its severity after cancer treatment. Measurement of serum biomarker, such as troponin, is valuable for detection of early signs of cardiotoxic effect during chemotherapy [29]. Biomarker analysis is quite simple and accurate for diagnosis and severity assessment of cardiotoxicities. When the biomarker studies suggest possible cardiac abnormalities associated with ECG abnormalities, non-invasive imaging study should be performed in the next step for precise analysis. Long-term follow-up imaging is essential to identify delayed cardiotoxic effects and to monitor cardiac function over time. Imaging biomarkers may provide a way to diagnose toxicity before the development of irreversible cardiovascular damage, particularly in the early stage of cancer therapy.

The choice of imaging modality in cardio-oncology is influenced by several factors, including the patient’s clinical condition, the specific information needed, the availability of imaging technology, and the overall treatment plan. Different cancer treatments have varying cardiotoxic profiles. For instance, anthracyclines are known to cause left ventricular dysfunction, while radiation therapy can lead to myocardial fibrosis and coronary artery disease. The imaging modality should be selected based on the expected cardiotoxicity profile of the treatment.

The imaging may also lead a way of care that allows cancer patients to continue their treatment safely without major cardiac events. There are number of evidence showing importance of monitoring left ventricular function during cancer therapy. However, the characteristics and clinical evidence of right ventricular impairment are rather poorly described [30]. In addition, vascular inflammation should be considered as one of the major risks after cancer therapy. Furthermore, thromboembolic complications leading to an increased risk of pulmonary embolism is often observed, particularly after treatment with tyrosine kinase inhibitors and serine–threonine kinase inhibitors. Thus, imaging analysis should play important roles for assessing cardiovascular functions after cancer therapy. Characteristics of imaging techniques for identifying cardiotoxicity are summarized in Table 1 [31, 32].

**Table 1:** Characteristics of imaging techniques for identifying cardiotoxicity

Imaging modalities	Parameters	Advantages	Disadvantages
Echocardiography	LVEF, GLS, RV/LV diastolic function	Easy & real time assessment Reliable LV/RV function Anatomical assessment No radiation	Less reproducible for patients with poor acoustic window Operator dependent
CT angiography	Coronary calcification Coronary stenosis CT-FFR	Excellent CV risk analysis Excellent anatomical study for Coronary arteries and pericardium	Limited by high heart rate, arrhythmias, or severe calcification
Cardiac MRI	T1/T2 mapping ECV Global/circumferential strain	Precise assessment of edema, fibrosis, & inflammation Assessment of pericardium No radiation	High cost
SPECT perfusion study	Fibrosis, perfusion	Ischemia and fibrosis detection	Relatively high cost Limited accessibility
PET perfusion study	Myocardial blood flow Myocardial flow reserve	Detection of micro-vascular abnormalities Quantitative assessment	High cost Limited accessibility
FDG-PET	Glucose metabolism Macrophage function	Detection of active Inflammation Viability study	High cost Limited accessibility
New molecular imaging	MIBG (sympathetic function) BMIPP (fatty acid uptake) DOTATATE (macrophage infiltration) FAPI (remodeling)	Quantitative analysis of various molecular functions	High cost Limited accessibility Limited data base in the new PET radiotracers

LVEF = left ventricular ejection fraction, GLS = global longitudinal strain, RV = right ventricle, LV = left ventricle, ECV = extracellular volume, FFR = fractional flow reserve, CV = cardiovascular,

FDG = ^18^F-fluorodeoxyglucose, PET = positron emission tomography,

MIBG = ^123^I-metaiodobenzyl gluanidine, BMIPP = ^123^I- beta-methyl-iodophenyl pentadecanoic acid DOTATATE = ^68^ Ga-DOTATATE, FAPI = ^68^ Ga-fibroblast-activating protein inhibitor

Echocardiography has been used as a gold standard in cardio-oncology field since this is easily performed. Echocardiography is excellent for assessing cardiac function, including left ventricular ejection fraction (LVEF), diastolic function, and myocardial strain. LVEF is often used as the main parameter for detecting changes in left and right ventricular function. Particularly, advanced assessment of right ventricular function by 3D-echocardiography is valuable for cardiotoxicity analysis [33]. New imaging techniques such as longitudinal strain on echocardiography, cardiac MRI, and nuclear imaging have recently been focused on this field. Myocardial strain is expected to be used for detecting early changes before cardiac function deteriorates showing better interobserver agreement than LVEF. Recent meta-analysis study indicated good prognostic performance of global longitudinal strain for subsequent LV dysfunction from anthracycline therapy [34]. Thus, strain analysis has the most benefit for patients with low-normal LVEF, suggesting as a new and sensitive marker for closer surveillance and possible cardio-protection during and after cancer treatment [35].

While echocardiography is easy and most commonly performed for monitoring biventricular function, it is often difficult to assess function for those with poor acoustic window. In addition, various functional parameters seem to be rather operator dependent.

Cardiac CT is a standard test for the diagnosis of atherosclerotic cardiovascular disease. High resolution and reduced radiation exposure permits wide applications in various patients suspected with cardiovascular disease. For cardio-oncology cardiac CT provides precise risk assessment in various cancer patients. Cardiac CT is valuable for assessing structural abnormalities of myocardium, coronary arteries, and aorta [36]. It provides detailed images of the heart's structures, including the heart chambers, valves, and major vessels. This is important for evaluating patients who might have valvular heart disease, especially if related to previous cancer treatments like radiation therapy. Non-contrast CT scanning is employed to assess the amount of calcium in the coronary arteries, serving as a dependable indicator of cardiovascular risk. Coronary CT is widely performed for those suspected with coronary artery disease. It also utilized to rule out coronary artery stenosis in patients who have undergone cardiotoxic cancer treatments and have subsequently shown a decrease in LVEF [36]. In addition, myocardial fibrosis and arteritis which is often seen after radiotherapy is well identified [37].

Cardiac CT shows an extremely high negative predictive value for high-risk patients. However, the diagnostic accuracy for cardiovascular disease by cardiac CT is limited for those with higher heart rate, arrhythmias or severe calcification [37, 38].

Cardiac MRI has been used as the reference standard for the measurement of cardiac chamber volumes, myocardial mass, and contractile function. It may identify structural alteration such as biventricular dysfunctions and functional changes in the myocardium, including signs of edema and inflammation, possibly prior to the left ventricular dysfunction [37, 38]. From a functional standpoint, minor initial changes in tissue post-chemotherapy can lead to localized impairments in wall motion as an early indicator of heart toxicity, identifiable through strain-based cardiac MRI [39]. Cardiac MRI is useful to identify perfusion defects with similar diagnostic value with CT perfusion and radionuclide perfusion imaging. The main advantage of cardiac MRI is myocardial tissue characterization by using multiparametric imaging, such as late gadolinium enhancement to identify gross scar, T1 mapping to define interstitial fibrosis and extracellular volume (ECV) increase, and T2 mapping to assess myocardial edema. Myocardial edema and increased ECV are the earliest surrogate markers of chemotherapy or radiation [36]. Several research works have shown that patients who received cardiotoxic chemotherapy exhibit an increased ECV compared to a similar group of individuals who did not undergo such treatment [40]. ECV is considered as an indirect biomarker of tissue fibrosis and interstitial space expansion [36]. An increase in pre-contrast T1 is associated with myocardial edema, inflammation, and fibrosis [41]. On the other hand, an increase in T2 relaxation time is associated with acute myocardial edema, as a marker of water-sensitive process [42]. Generally, cardiotoxic drug exposure has been associated with an increase in both native T1 and T2 relaxation time [43]. A number of studies suggested valuable role of increased T2 relaxation time as an early acute sign of cardiotoxicity [44–47].

These changes are often observed with various cardiotoxic drug therapies as well as radiotherapy. MRI permits semiquantitative assessment of tissue characterization. In addition, there is no radiation burden to the patients. Therefore, both baseline study before the treatment and follow-up study after the treatment are valuable [47–49]. For screening and monitoring of cardiac function in patients with cancer treated with cardiotoxic chemoradiotherapy, cardiac MRI is considered as a second-line modality after echocardiography, particularly for those with difficult sonographic window [49]. The role of cardiac MRI in cardio-oncology continues to grow.

## Nuclear imaging in cardio-oncology

Nuclear medicine imaging has also been commonly applied for quantitative assessment of cardiovascular function after cancer therapy [50–53]. LVEF is accurately assessed with high reproducibility using ECG-gated blood pool scan and ECG-gated perfusion imaging as well. Myocardial perfusion imaging using single-photon emission CT (SPECT) and positron emission tomography (PET) have been applied for many patients suspected with coronary artery disease. SPECT is often used for assessing myocardial ischemia. SPECT perfusion study offers number of advantages over echocardiography or MRI, including applications for every patient such as obesity, metal device, or kidney failure with low intra- and interobserver variability [54]. PET perfusion study, using ^15^O-water, ^82^Rb, ^13^N-ammonia, or new pharmaceutical agent ^18^F-flurpiridaz, is valuable for quantitative analysis of myocardial blood flow and myocardial flow reserve [54–56]. Such quantitative assessment of myocardial blood flow and flow reserve by PET hold a promise for precise assessment of disease mechanism and its severity after cancer therapy [57]. Particularly, ischemia with non- obstructive coronary artery (INOCA) has recently been focused for risk analysis after cancer therapy. Nuclear perfusion study, particularly by PET, has an important role for accurate diagnosis of INOCA and risk analysis in various cardiovascular diseases [57, 58].

Molecular imaging using MRI and nuclear imaging are used for assessing tissue function before and after cancer therapy on molecular perspectives. Values of tissue characterization by MRI has been described previously. MRI is particularly valuable for monitoring cardiovascular dysfunction due to no radiation as compared to nuclear imaging. Cardiac MRI provides detailed information on cardiac anatomy and can detect myocardial fibrosis, edema, and infarction. On the other hand, nuclear imaging should play an important role for assessing cardiotoxicity using various radionuclide tracers. PET with ^18^F-fluorodeoxyglucose (FDG) has been used as a marker of glucose utilization. FDG uptake may reflect oxidative stress and alterations in cardiac metabolism. In particular, an increase in FDG accumulation is associated with an active inflammatory process, allowing for the activity of the inflammatory disease to be evaluated under fasting condition [59]. Therefore, FDG-PET is valuable for detecting active cardiovascular inflammation, particularly in the early stage of cancer therapy and monitoring toxic effects [52, 60, 61]. Cardiovascular toxicity may carry a risk of arterial thrombosis, including myocardial infarction. FDG serves as a sensitive indicator of the metabolic shift in the myocardium, which occurs in the early stages of coronary artery disease [53]. Distinct patterns of FDG uptake, particularly in the right ventricle, have been associated with anthracycline cardiotoxicity [62]. FDG uptake may not be specific for cancer-related inflammation but also for ischemic but viable myocardium, and therefore, careful prolonged fasting with food control is required for suitable FDG-PET study [59]. FDG-PET has been used for identifying ischemic myocardium, active myocarditis, and vasculitis after chemotherapy, immunotherapy, and radiation therapy.

**Fig. 1:**
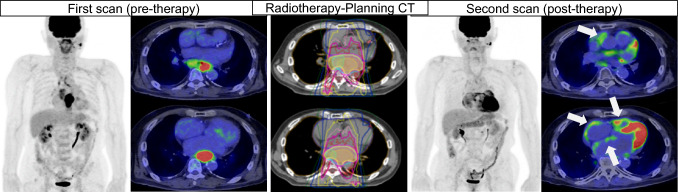
FDG-PET/CT scan before chemo-radiotherapy (left), radiotherapy planning CT (middle), and FDG-PET/CT scan after chemo-radiotherapy (right) in a patient with esophageal cancer with lymph node involvement. High FDG uptake in mid-esophagus and surrounding lymph nodes is noted before the treatment, which disappears after the treatment. New FDG uptakes are observed in the right ventricle and atrium suggesting radiation-induced myocarditis and increased afterload after the treatment. (white arrows)

FDG-PET plays a crucial role in not only oncology but also cardio-oncology fields due to its ability to visualize metabolic activity in the whole-body. FDG-PET is commonly used for oncology studies showing localization and extension of cancer throughout the body. This imaging particularly important for assessing treatment strategy and treatment effect as well with various anticancer therapy. Therefore, FDG-PET after cancer therapy is considered as an elegant approach for simultaneous assessment of tumor response to cancer therapy and possible cardiovascular dysfunction as well [63] (Fig. 1).

The risk of vascular disease posed by the cancer itself is increased by cancer therapies. Vascular toxicities are the second most common cause of death in patients with cancer undergoing outpatient therapy [64]. Number of studies suggested chemotherapy-related vascular side effects and radiotherapy-related vascular side effects [64]. FDG-PET can demonstrate not only myocardial inflammation but also active vasculitis. Recent studies nicely suggested that FDG-PET/CT can identify unstable atherosclerosis and active vasculitis as focal FDG uptake [65, 66]. FDG-PET to apply for those with arteritis has recently been approved for insurance coverage in Japan [61]. While there remain limited clinical reports at present, FDG-PET should play an important role for identifying and managing active vasculitis after cancer therapy.

One of the single-photon molecular imaging biomarkers is ^123^I-meta-iodobenzylguanidine (MIBG), a radiolabeled norepinephrine analogue. Cardiac neuronal function is compromised in various cardiac diseases, such as heart failure, ischemia, arrhythmia, and some types of cardiomyopathy [67]. Functional and structural injury to myocardial adrenergic neurons may also be accompanied with the pathophysiology of cancer therapy-related cardiotoxicity. Thus, this is a novel approach for imaging dysregulated presynaptic norepinephrine homeostasis as a prognostic heart failure. The myocardial MIBG imaging can demonstrate dysregulated presynaptic norepinephrine homeostasis which is used as a prognostic marker in heart failure [68–71]. Cardiac innervation imaging is preferable, which may be an early cardiotoxicity marker before decrease in LVEF occurs. Recent report suggests increasing MIBG washout as a marker for myocardial compensation to cardiotoxic injury from anthracycline exposure [72].

Another molecular imaging biomarker is ^123^I-beta-methyl-iodophenyl pentadecanoic acid (BMIPP) as a marker of fatty acid uptake in the myocardium which is commonly used in Japan [73–75]. Focal decrease in BMIPP uptake in the myocardium is commonly observed after radiation therapy, suggesting myocardial damage or fibrosis [76, 77]. The combined imaging analysis using FDG-PET and BMIPP SPECT indicated focal damage with metabolic alteration in the myocardium after radiotherapy in esophageal cancer [78].

There are a few other PET molecular imaging biomarkers applied for early assessment of cardiotoxicity. Somatostatin receptor PET using ^68^ Ga-DOTATOC/DOTATATE is new established method for staging or restaging of patients with neuroendocrine tumors. In addition, this tracer can visualize myocardial macrophage infiltration [79]. This visualization permits early detection of myocardial inflammation in patients with pericarditis, myocarditis, or subacute myocardial infarction and serve as a potential predictor of cardiac remodeling processes [79]. Recent reports describe new applications of somatostatin receptor PET in immunotherapy-induced myocarditis [80]. One of the major advantages of this tracer is no requirement of diet control, which is mandatory in FDG study. Note that the synthesis of ^68^ Ga tracers requires a Ge/Ga generator but not in-house cyclotron, which could be an advantage in some regions. More recently, ^18^F- or ^68^ Ga-labeled fibroblast activation protein inhibitor (FAPI) tracers have attentions since it also enables simultaneous assessment for tumor activity and cardiac diseases (e.g., myocardial infarction and atherosclerosis) [81].

## Future perspectives

Cardio-oncology represents an important new area that should be covered by multiple specialist teams, including medical and radiation oncologists, cardiologists, diagnostic radiologists, technologist, nurses, and pharmacists. Interdisciplinary cooperation among these specialists is mandatory for accurate and timely diagnosis and also suitable management for each cancer patient. Cardiologists should be knowledgeable about the strengths and limitations of various imaging modalities, including echocardiography, cardiac MRI, and nuclear imaging. They should select the appropriate modality based on the clinical scenario and the specific information needed. Many oncologists should understand various cardiotoxic effects after new cancer treatments. In addition, radiologists should play an important role for selecting suitable imaging modalities and interpreting not only the tumor itself but also the condition of the heart when viewing tumor images in order to provide important messages from these images for both cardiologists and oncologists. Radiologists should be aware of the critical role that imaging plays in the diagnosis, monitoring, and management of cardiotoxicity in cancer patients. Understanding the appropriate use of each imaging modality is essential for providing comprehensive care. Radiologists should stay informed about the latest advancements in imaging technologies and techniques, such as cardiac MRI and nuclear imaging, to ensure accurate assessment and timely detection of cardiotoxic effects.

Cardiovascular imaging has evoked as a key role for this purpose, allowing non-invasive evaluation of cardiovascular alterations complimentary to biomarkers and clinical assessment. Suitable imaging selection and interpretation may permit not only for early diagnosis of cardiovascular injury but also accurate assessment treatment effects. Thus, the imaging will provide impact on therapeutic management and improved outcome after cancer therapy. Future studies are warranted to assess the promising potential of these non-invasive cardiovascular imaging in cardio-oncology.
